# The influence of neighborhood built environment on school-age children’s outdoor leisure activities and obesity: a case study of Shanghai central city in China

**DOI:** 10.3389/fpubh.2023.1168077

**Published:** 2023-06-27

**Authors:** Weifan Tan, Xiaocong Lu, Tingting Xiao

**Affiliations:** ^1^School of Sociology, Shanghai University, Shanghai, China; ^2^School of Physical Education, Shanghai Normal University, Shanghai, China

**Keywords:** social ecology theory, neighborhood built environment, outdoor leisure activities, obesity, school-age children

## Abstract

**Objective:**

The aim of this study was to examine the influencing pathways of the neighborhood built environment on children’s outdoor leisure activities and obesity.

**Methods:**

A total of 378 elementary school students from 10 schools in central Shanghai were selected by a convenient sampling method for questionnaire survey and accelerometer tracking.

**Results:**

1) The neighborhood built environment could affect children’s obesity not only through direct effect (*β* = 0.15, *p* < 0.05), but also through the mediating effect of outdoor leisure activities (*β* = 0.19, p < 0.05). 2) For boys, the neighborhood built environment could affect children’s obesity not only through direct effect (*β* = 0.17, *p* < 0.05), but also through the mediating effect of outdoor leisure activities (*β* = 0.26, *p* < 0.05). For girls, the neighborhood built environment could affect children’s obesity only through the mediating effect of outdoor leisure activities (*β* = 0.13, *p* < 0.05).

**Conclusion:**

The neighborhood built environment and outdoor leisure activities are important influencing factors in children’s obesity. The neighborhood built environment and outdoor leisure activities could have direct and indirect effects on children’s obesity, while there are gender differences in the influencing pathways of the neighborhood built environment on children’s obesity. This study suggests that improving the neighborhood built environment and promoting outdoor leisure activities in children have important value for influencing children’s obesity.

## 1. Introduction

The Blue Paper of the China Child and Adolescent Nutrition and Health Report 2016 points out that, from 1985 to 2014, the detected rate of obesity among students in China increased sharply, and the risk of chronic diseases such as cardiovascular and hyperglycemia due to obesity subsequently increased (1). The Healthy China Initiative (2019–2030) also points out that the rate of students’ obesity meeting the standard was only 31.8% in 2017, and the obesity problem has become a serious health threat to school-age children and adolescents in China (2). Providing a supportive environment for children and encouraging them to participate in diverse outdoor leisure activities are important means to promote physical activity and reduce their obesity, and are also an important entry point for the construction of child-friendly cities.

Social ecological theory suggests that individual health is influenced by the interaction of multiple factors such as individual factors (cognition, attitude, self-efficacy, etc.), interpersonal factors (peer support, parental support, etc.), environmental factors (parks, green spaces and recreational facilities, etc.), and policy. It has also been confirmed that obesity is closely related to geography, and geographic research on the relationship between human health and geography has gradually turned to the influence of elements of the urban built environment on human health (3–5). According to the spatio-temporal model of children’s activity domain proposed by Matthews, children’s daily activities are centered on the home and expand outward in a circle-like manner from familiar and habitual areas (6). Studies have shown that 63% of children’s physical activities occur within the vicinity of their homes (7); the results of studies on the characteristics of children’s outdoor activities in China also show that children’s outdoor activities mainly occur in and around residential areas (8). The neighborhood built environment, as a spatial carrier carrying children’s life and growth, accommodates most of the daily activities of children and is an important social determinant of children’s health.

There is abundant international research on the relationship between the built environment and children and adolescents’ outdoor activities and their health, and studies have found that built environment factors such as the commuting distance to and from school, greenery around residential neighborhoods and schools, intersection density, open space distribution, and access to outdoor sports fields and facilities can have significant effects on the outdoor activities of children and adolescents (9–11). In addition, a large number of studies have confirmed that the urban built environment is significantly associated with the healthy development (obesity) of children and adolescents, including factors such as the residential density, road density, neighborhood safety, and recreational facilities; all of these can significantly affect children and adolescents’ outdoor activities and obesity (12, 13). At the same time, there are significant gender difference in this correlation (14). Currently, research in the field of the built environment and physical activity in China has mainly focused on the effects of microscopic activity venues, facility configuration, and design on the physical activity and health of elders (15–17), with little attention dedicated to school-age children (18). Accurate identification of the influencing factors of children’s obesity and their acting pathways could provide both theoretical support for promoting the healthy development of children and adolescents and guidance for the effective prevention and intervention of obesity in children and adolescents.

**Figure 1:**
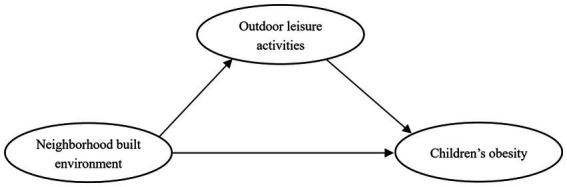
Hypothetical path model.

Previous studies mostly examined the relationship between two variables in the built environment: physical activity and obesity. This study simultaneously placed three variables in one model, to examine the influencing pathways of the neighborhood built environment on children’s outdoor leisure activities and obesity, which is an important supplement to the research in this field. This study used children’s obesity as the dependent variable and the neighborhood built environment as the explanatory variable. With children’s outdoor leisure activities as the mediating variable, the factors influencing children’s obesity were grouped into a hypothetical theoretical model (as in Figure 1). It was hypothesized that the neighborhood built environment is an important factor determining children’s outdoor leisure activity, which in turn influences their obesity, and that the neighborhood’s built environment can either directly influence children’s obesity or indirectly influence children’s obesity through outdoor leisure activities; moreover, the influence path may also have gender differences.

## 2. Materials and methods

### 2.1. Data collection

**Figure 2:**
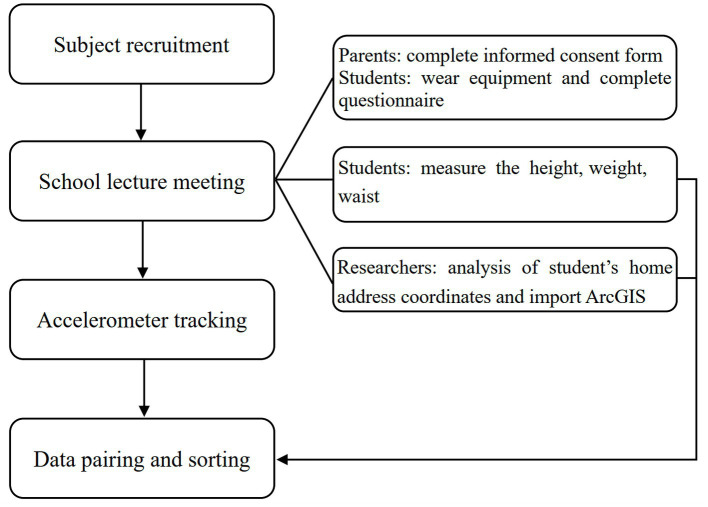
Research flowchart.

There are 10 administrative regions in Shanghai central city, and randomly select a primary school in each administrative region to conduct a meeting on the recruitment of subjects. All primary school students voluntarily participated in the meeting and signed up for this study. A total of 378 ordinary primary school students were recruited from 10 primary schools in Shanghai central city for the survey. The economic level of the central city is higher than that of the non-central city, and this area has the highest house prices in Shanghai, so the family economic statuses of children involved in this study is generally high. All surveys were completed from November 2 to 30, 2020. Before the survey, the school was the unit used to recruit the test subjects and hold a meeting for the subjects. At the meeting, we guided them on how to fill in the questionnaire and wear measuring equipment, as seen in the research flowchart (Figure 2). The questionnaire content includes gender, age, grade, home address, height, weight, waist circumference, etc. This study was approved by the Institutional Review Board of Shanghai University. Written informed consent was obtained from the adolescents involved in the study and their parents or guardians.

### 2.2. Variables

#### 2.2.1. Neighborhood built environment

The neighborhood built environment variables investigated included density, diversity design, and accessibility. The density involved two variables: population density and building density. The diversity design involved three variables: street connectivity, *per capita* road length, and mixed land utilization rate. The accessibility involved four variables: the number of traffic stations, distance to a traffic station, distance to a fitness facility, and distance to a commercial area (19). Geographic information system (GIS; ArcGIS 10.2, Environmental Systems Research Institute, RedLands, America) technology was used to collect the neighborhood built environment variables. The full-element digital map and vector map of the downtown streets of Shanghai were imported into ArcGIS 10.2 to obtain the spatial data. The spatial data included various fitness and leisure facilities, public places, the road network, the rail transit network, etc. Based on the radius of public facilities (such as bus stops and fitness trails) in Shanghai and the division of urban public sports space proposed by Cai et al. (20), the buffer radius was set at 1500 m. A buffer zone with a range of 1,500 m was generated with the participants’ residential area as the center. Next, clip processing was performed on the buffer zone to extract the data on the built environment variables.

#### 2.2.2. Outdoor leisure activities

Children’s outdoor leisure activities were obtained using an ActiGraphGT3X+ accelerometer. It was found that the built environment is not a significant predictor of children and adolescents’ physical activity on weekdays but a significant predictor on weekends (19). Therefore, the variables selected for this study were children’s outdoor leisure activity on weekends, specifically the mean value of children’s moderate-to-high-intensity outdoor leisure activity on weekends, the amount of outdoor leisure activity of children on weekends, and the duration of adolescents’ outdoor leisure activity on weekends. The initialization of the device was completed before the survey, and a briefing session was conducted to instruct the subjects to put on and take off the survey device and confirm the wearing time (Saturday and Sunday, 7:00 ~ 21:00, except for bathing, swimming, and sleeping) and wearing site (right hip). After the survey, the survey devices were returned to the research team for unified retrieval. The ActiGraph GT3X^+^ parameters are shown in Table 1.

**Table 1:** ActiGraph GT3X^+^ parameters.

	Parameter	Details
1	Monitor	ActiGraph GT3X^+^
2	Sampling frequency	30 Hz
3	Sampling interval	5 s
4	Wearing time	≥480 min/24 h
5	Number of days of data collection required for valid data collection	≥3 days on weekdays and one day on weekends
6	Physical activity intensity levels	Low: 100–1,679 counts per minute (CPM)
		Moderate-to-vigorous: 1,680–3,368 CPM
		High: >3,368 CPM

#### 2.2.3. Obesity

Children’s obesity was evaluated using a combination of body mass index and waist-to-height ratio. The body mass index is an important standard that is commonly used internationally to measure the degree of obesity and healthiness of the human body; it is also an important indicator in the national obesity standards for students. In addition, the study also chose the index of waist-to-height ratio to jointly evaluate the obesity of adolescents. A study developed by Sun et al. compared the consistency of the body mass index and waist-to-height ratio in judging the obesity of adolescents and found that judging the obesity of adolescents using the waist-to-height ratio combined with body mass index was better than judging it using a single method (21). In our study, trained researchers went to the school and performed all of the measurements on all of the children at the school meeting. Due to the large age span of school-age children, to make the data more comparable, we standardized the BMI and WHR and obtained the new standardized scores: body mass index z-score (zBMI) and waist-to-height ratio z-score (zWHR) (22).

### 2.3. Statistical analysis

The neighborhood built environment, leisure physical activity, and obesity were obtained by measuring variables, respectively, forming three measurement models. The structural relationship of the three potential variables established by the three measurement models forms a structural model, and the structural factor relationship of the potential variables was discussed with the strategy of path analysis. This study conducted validating factor analysis using SPSS 22.0 software and then constructed structural equation models using AMOS 24.0 software to calculate the relationship of each potential variable and validate the theoretical model proposed by the study. The observed indicators of the neighborhood built environment include 9 items, such as the population density, street connectivity, number of traffic stations, etc.; the observed indicators of children’s outdoor leisure activities are the time spent on medium-to-high-intensity outdoor leisure activities on weekends, total count value, and total activity time. A structural equation model was used to verify the relationship between the neighborhood built environment, outdoor leisure activities, and children’s obesity. Using mean to fill in missing data during data processing and analysis. Alpha level used *p* < 0.05. The multivariate fit index was used to evaluate the pathway model of the neighborhood built environment affecting children’s obesity, and the evaluation indicators were χ2 /df, RMSEA, RMR, NFI, CFI, GFI, and AGFI (23).

## 3. Results

A total of 400 questionnaires were distributed for the study; 391 were collected, 380 valid data were recovered by accelerometers, and 378 valid data were obtained by complete matching of questionnaires with accelerometer data. The basic situation of the subjects is shown in Table 2.

**Table 2:** Basic information about the subjects.

Categories		Number (%)	Age (M ± SD)
Gender	Boys	195 (51.59)	9.11 ± 1.20
	Girls	183 (48.41)	9.32 ± 1.09
Grade	Grade one	72 (18.78)	6.58 ± 1.21
	Grade two	74 (19.58)	7.44 ± 0.98
	Grade three	79 (20.90)	8.63 ± 1.31
	Grade four	77 (20.63)	9.27 ± 0.98
	Grade five	76 (20.11)	10.48 ± 1.11

As shown in Table 3, the descriptive statistics of the study variables showed that the mean the BMI value was 20.02 for boys and 19.97 for girls, which are normal grades on the scale for each grade in the National Physical Fitness Standards for Students. According to the critical value point of abdominal obesity for boys (0.47) and girls (0.45), which was delineated by Zhou et al. based on the data of 16,914 children and adolescents in six provinces and cities in China (24), it can be found that, in the waist-to-height ratio index, the values of the waist-to-height ratio of boys and girls in this study did not reach the threshold value of abdominal obesity, but boys those of were slightly higher than girls (0.46 > 0.42). Meanwhile, the standard deviation of the waist-to-height ratio for boys and girls was relatively large, indicating that the dispersion degree of children’s waist-to-height ratio was relatively large. Among the indicators of outdoor leisure activities, the length of moderate-to-vigorous outdoor leisure activity was 70.21 min/day for boys and 60.92 min/day for girls, which is basically in line with the standard recommended by the Guidelines for Physical Activity for Children and Adolescents in China, which stipulates that children and adolescents should engage in at least a total of 60 min of daily moderate to vigorous physical activity (25).

**Table 3:** Descriptive analysis of variables.

Variables			Boys (M ± SD)	Girls (M ± SD)	Total (M ± SD)
Obesity		Body mass index	20.02 ± 2.83	19.97 ± 2.34	20.00 ± 2.66
		Waist-to-height ratio	0.46 ± 0.45	0.42 ± 0.54	0.45 ± 0.50
Neighborhood built environment	Density	Population density (number/km^2^)	56,174 ± 20,865	55,234 ± 21,174	55,801 ± 20,977
		Building density (%)	0.53 ± 0.18	0.55 ± 0.20	0.54 ± 0.19
	Diversity design	Street connectivity (number/km^2^)	23.24 ± 7.15	23.18 ± 6.98	23.20 ± 7.11
		*Per capita* road length (m)	0.26 ± 0.47	0.27 ± 0.46	0.26 ± 0.46
		Mixed land utilization rate	14 ± 2	15 ± 3	14 ± 3
	Accessibility	Number of traffic stations	7 ± 3	8 ± 2	8 ± 2
		Distance to traffic station (m)	211.34 ± 100.98	208 ± 102.45	210.61 ± 101.24
		Distance to fitness facility (m)	135.23 ± 121.62	130.99 ± 109.87	133.67 ± 110.78
		Distance to commercial area (m)	350.11 ± 221.54	361.05 ± 210.55	355.52 ± 215.84
Outdoor leisure activities		Moderate-to-vigorous physical activity (min/day)	70.21 ± 20.45	60.92 ± 17.78	64.43 ± 20.07
		Total count	278,476.24 ± 32,562.47	193,447.81 ± 56,432.52	240,811.63 ± 45,747.48
		Total activity time (min)	285.52 ± 100.44	254.17 ± 101.35	265.28 ± 100.24

**Figure 3:**
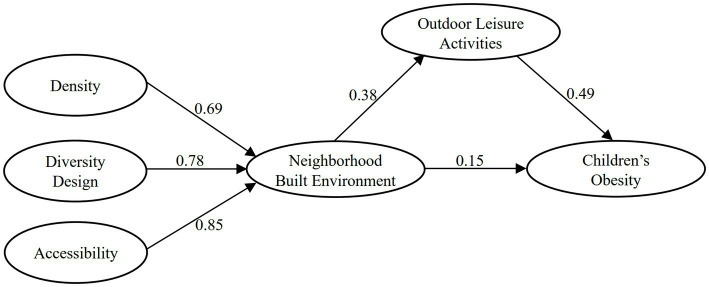
A pathway model of neighborhood built environment influencing children’s obesity. This is a simplified model, where the solid lines represent significance and the normalized path coefficients are labeled.

The modified model fit results in this study were: χ^2^/df = 2.05, RMSEA = 0.07, RMR = 0.04, NFI = 0.97, CFI = 0.95, GFI = 0.98, and AGFI = 0.96; All indicators were within the standard range, indicating that the pathway model of the neighborhood built environment affecting children’s obesity fits well and that the theoretical model was acceptable. As shown in Figure 3, according to the path model of the neighborhood built environment affecting children’s obesity: 1) the neighborhood built environment comprises three dimensions—density, diversity design, and accessibility—among which the density is measured by two observations, diversity design is measured by three observations, and accessibility is measured by four observations; 2) outdoor leisure activity is measured by three observations: the mean value of moderate-to-vigorous outdoor leisure activity, amount of outdoor leisure activity, and duration of outdoor leisure activity; 3) children’s obesity is measured by two variables: body mass index z-score and waist-to-height ratio z-score. The relationship between the potential variables reflected in the path model shows the following: 1) The direct effect of the neighborhood built environment on children’s obesity is 0.15 (CI [0.08, 0.27]), the indirect effect of the neighborhood built environment on children’s obesity through outdoor leisure activities is 0.38 × 0.49 = 0.19 (CI [0.01, 0.10]), and the overall effect of the neighborhood built environment on children’s obesity is 0.15 + 0.19 = 0.34 (CI [0.13, 0.32]). The direct and indirect effects of the neighborhood built environment on children’s obesity are statistically significant at the 0.05 level, indicating that neighborhood built environment could direct affect children’s obesity and also indirect affect it through outdoor leisure activities. The indirect effect of the neighborhood built environment on children’s obesity is greater than the direct effect, indicating that the effect of the neighborhood built environment on children’s obesity was mainly achieved through the indirect effect of outdoor leisure activities.

**Figure 4:**
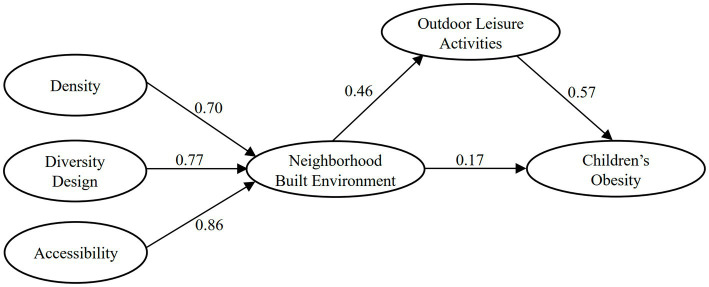
The pathway model of the neighborhood built environment affecting boys’ obesity. This is a simplified model, where the solid lines represent significance and the standardized path coefficients are labeled.

**Figure 5:**
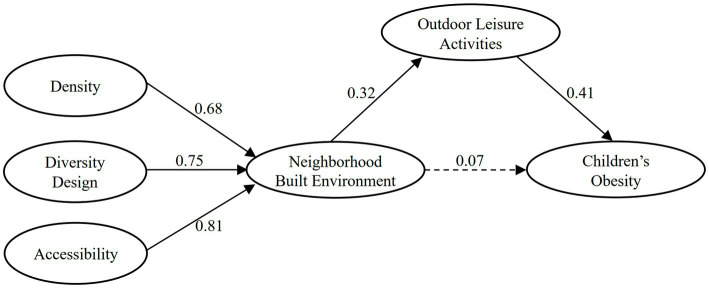
The pathway model of the neighborhood built environment affecting the girls’ obesity. This is a simplified model, where the solid line represents significance, the dashed line represents insignificance, and the standardized path coefficients are labeled.

To examine the gender differences in the influence of the neighborhood built environment on children’s obesity, this study constructed pathway models to determine the influence of the neighborhood built environment on boys’ obesity (Figure 4) and girls’ (Figure 5) obesity. As above, multivariate fit indicators were also used to evaluate the pathway models of the urban built environment affecting boys’ and girls’ obesity, respectively. The results demonstrated that: 1) the fitting indicators for the pathway model of the neighborhood built environment affecting boys’ obesity were: χ^2^/df = 2.50, RMSEA = 0.06, RMR = 0.04, NFI = 0.96, CFI = 0.95, GFI = 0.97, and AGFI = 0.95; All indicators are within the standard range, indicating that the pathway model of the neighborhood built environment affecting the boys’ obesity fits well and the model is acceptable; 2) The fitting indicators for the pathway model of the neighborhood built environment affecting girls’ obesity were: χ^2^/df = 2.48, RMSEA = 0.07, RMR = 0.03, NFI = 0.95, CFI = 0.96, GFI = 0.94, and AGFI = 0.97; All indicators were within the standard range, indicating that the pathway model of the neighborhood built environment affecting girls’ obesity also fits well and the model is acceptable.

As shown in Figure 4, using the path model of the neighborhood built environment affecting boys’ obesity, we can find that the direct effect of the neighborhood built environment on boys’ obesity is 0.17 (CI [0.21, 0.35]), the indirect effect of the neighborhood built environment on boys’ obesity through outdoor leisure activities is 0.46 × 0.57 = 0.26 (CI [0.05, 0.14]), and the overall effect of the neighborhood built environment on boys’ obesity is 0.17 + 0.26 = 0.43 (CI [0.30, 0.41]). Both the direct and indirect effects of the neighborhood built environment on boys’ obesity were statistically significant at the 0.05 level, indicating that the neighborhood built environment could direct affect children’s obesity and through outdoor leisure activities indirect affect children’s obesity. As shown in Figure 5, the path model of the neighborhood built environment affecting girls’ obesity revealed that the direct effect of the neighborhood built environment on girls’ obesity is 0.07 (CI [−0.13, 0.02]) but is not statistically significant at the 0.05 level, meanwhile the indirect effect of the neighborhood built environment on girls’ obesity through outdoor leisure activities is 0.32 × 0.41 = 0.13 (CI [0.21, 0.34]) and is significant at the 0.05 level, indicating that the neighborhood built environment could only affect girls’ obesity through the indirect effect of outdoor leisure activities. It was found that there are gender differences in the pathways by which the neighborhood built environment affects children’s obesity; for boys, the neighborhood built environment could direct affect their obesity and through outdoor leisure activities indirect affect their obesity, while for girls, the neighborhood built environment could only indirect affect their obesity through outdoor leisure activities.

## 4. Discussion

This study found that the neighborhood built environment plays an important role in reducing children’s obesity and that it can directly affect children’s obesity. Density, diversity design, and accessibility are important factors of the neighborhood built environment that affect children’s obesity. Many domestic and international studies have shown that the correlation between the urban built environment and children and adolescents’ obesity is significant. Studies from the United States have shown that the neighborhood environment is the second most important factor affecting the obesity of residents after individual characteristics (26). Based on survey data of 1,265 Chinese students, Wong et al. found that the neighborhood environment has a significant correlation with student obesity, and this is especially evident because students who lived in neighborhoods far from parks were more likely to be obese, i.e., the likelihood of school-age children being obese was positively correlated with their closest distance to nearby parks (27). Moreover, the scholar De-Bont also suggested that destination accessibility could effectively prevent the prevalence of obesity (28). In the current state of research, the results of domestic and international studies involving built environment accessibility elements have achieved consensus that the accessibility of surrounding facilities in a community neighborhoods could prevent adolescent obesity. In terms of street facility configuration, An et al. used a systematic literature review and found that the built environment has a significant impact on childhood and adolescent obesity in China, as well as that residential and surrounding sidewalks and parks are all relevant to the healthy development of children and adolescents (29).

This study found that the neighborhood built environment can also indirectly influence children’s obesity through the mediating role of outdoor leisure activities. The indirect effect accounted for 55.56% of the total effect, indicating that the neighborhood built environment plays a positive antecedent role in reducing children’s obesity and the mediating role through outdoor leisure activities is the main pathway through which the neighborhood built environment influences children’s obesity. At present, the academic community has basically reached a consensus on the relationship between physical activity and obesity, especially on the important role of physical activity in suppressing childhood and adolescent obesity; Moreover, domestic and foreign scholars have reached a high degree of agreement on physical activity’s ability to suppress adolescent obesity. Thus, how to promote and increase the physical activity level of children and adolescents is the main direction to curb childhood and adolescent obesity. In terms of accessibility, it has been found that accessibility to public facilities is positively associated with physical inactivity among children and adolescents and that accessibility to sports venues has a positive effect on physical activity levels (2, 30). In addition, the accessibility and availability of parks and green spaces have a significant positive impact on residents’ physical activity satisfaction; specifically, the shorter the distance and time to parks and green spaces, the higher the residents’ physical activity satisfaction. Further, the more availability of parks and green spaces, the higher the residents’ physical activity satisfaction, and the quality of residences and surrounding vegetation are also closely related to residents’ physical activity satisfaction (31). In terms of design diversity, children and adolescents without sidewalks near their residences are more likely to be physically inactive, and student physical activity levels are positively correlated with the availability of multiple alternative trails near their residences, whether in central urban or suburban areas; building more recreational fitness facilities in the community can also help children and adolescents maintain or increase their recreational physical activities (18). Overall, based on the general context of urban planning, through the rational layout of urban space, improving the accessibility and diversity of facility design and actively planning or renovating the community fitness environment to achieve neighborhood-built-environment-based physical activity interventions for children and adolescents are not only effective in promoting children and adolescents’ physical activity but also have far-reaching effects, and the neighborhood built environment plays an important role in children and adolescent health promotion.

In addition, gender differences in the pathways of the neighborhood built environment on children’s obesity were found. For boys, the neighborhood built environment not only directly influences their obesity but also indirectly influences their obesity through the mediating effect of outdoor leisure activities; however, for girls, the neighborhood built environment does not directly influence their obesity but only indirectly influences their obesity through the mediating effect of outdoor leisure activities. Thus, the analysis of variance revealed that boys’ obesity could be directly influenced by the urban built environment compared to girls, i.e., the neighborhood built environment is more likely to influence boys’ obesity. This is generally consistent with the results of existing studies. Hu et al. found that boys without open space near their homes are more likely to be physically inactive, and boys without walking paths near their homes are more likely to be overweight and obese (32). Huang et al. (33) also found that for boys, higher residential density results in longer screen time and is also positively associated with the amount of time boys spent online and playing video games; They also posited that increased screen time/sedentary time is also a major potential contributor to adolescent obesity. Based on a study of the relationship between BMI and walking and environmental factors around the residences of school-age children in Macau, Ho et al. found that walking convenience around the residence and community could significantly influence obesity in male school-age children, and in addition, accessibility to facilities around the residence and community of male school-age children could also promote walking and reduce the prevalence of obesity (14). In this study, the direct effect of the neighborhood built environment on girls’ obesity is insignificant. However, this does not mean that the neighborhood built environment has no effect on girls’ obesity; the reason for this may be caused by the inconsistency in the observed variables of the built environment selected by different authors and is related to the observed variables of the built environment selected in this study.

There are limitations and challenges to the present study. First, this study adopted a cross-sectional study design, which cannot accurately reflect the causal relationship between variables. In the future, follow-up investigation could be continued, and the causal relationship between variables could be further verified through longitudinal follow-up research. Second, many factors affect children’s obesity, and more research variables could be included in the future, such as the family economic level, parents’ education, etc., and more in-depth multivariate analyzes are also very necessary.

## 5. Conclusion

The neighborhood built environment could affect children’s obesity not only through direct effect, but also through the mediating effect of outdoor leisure activities. Among them, the mediating role of outdoor leisure activities is the main pathway through which the neighborhood built environment influences children’s obesity. In addition, there are gender differences in the pathways through which the neighborhood built environment influences children’s obesity. For boys, the neighborhood built environment could affect children’s obesity not only through direct effect, but also through the mediating effect of outdoor leisure activities, while for girls, the neighborhood built environment could affect children’s obesity only through the mediating effect of outdoor leisure activities. Overall, the neighborhood built environment is more likely to affect the obesity of boys.

Research suggests that the neighborhood built environment plays an important role in the healthy development of adolescents during the critical period of growth and development, as typified by the ancient Chinese expression that “Mencius’ Mother Moved Three Times.” Therefore, reducing children’s obesity can be achieved from the perspective of urban planning by improving the built environment of neighborhoods and promoting children’s participation in active outdoor leisure activities. The World Health Organization released the “Healthy Cities: Effective Approach to a Rapidly Changing World” in 2020, which explicitly proposes the promotion of healthy urban planning and design. This aligns with the viewpoint of this study that actively improves the built environment of cities to promote the healthy development of school-age children. In terms of density, it was appropriate to increase population density and building density, which could increase children’s social interaction and be beneficial to their physical and mental health. In terms of diversity design, increasing street connectivity, *per capita* road length, and mixed land utilization rate could have a beneficial impact on children’s health. In terms of Accessibility, the physical activity level of children could be improved and their health can be promoted by increasing traffic stops around residential areas, shortening the distance to traffic stations, fitness facilities and business districts. In addition, the relevant government departments should also pay attention to the different needs of different genders and different groups of people in urban planning and construction to consider all factors and conduct coordinated development.

## Data availability statement

The raw data supporting the conclusions of this article will be made available by the authors, without undue reservation.

## Ethics statement

The studies involving human participants were reviewed and approved by the Institutional Review Board of Shanghai University. The participants legal guardian/next of kin provided their written informed consent to participate in this study.

## Author contributions

WT and XL: conceptualization. WT: methodology, formal analysis, investigation, writing—original draft preparation, resources, supervision, and project administration. TX: validation. WT, XL, and TX: writing—review and editing. All authors contributed to the article and approved the submitted version.

## Funding

This research was funded by the School Sports Research Project of Shanghai, grant number HJTY-2017-C05.

## Conflict of interest

The authors declare that the research was conducted in the absence of any commercial or financial relationships that could be construed as a potential conflict of interest.

## Publisher’s note

All claims expressed in this article are solely those of the authors and do not necessarily represent those of their affiliated organizations, or those of the publisher, the editors and the reviewers. Any product that may be evaluated in this article, or claim that may be made by its manufacturer, is not guaranteed or endorsed by the publisher.
